# The Ten Second Triage Tool – a multi-disciplinary simulation-based field test to determine its speed, accuracy and practical on scene application

**DOI:** 10.1186/s13049-026-01588-3

**Published:** 2026-03-23

**Authors:** Claire Louise Park, James Vassallo, Philip Cowburn, Sean Brayford-Harris, Bryony Louise Dunne, Christopher G. Moran, Jason Edward Smith

**Affiliations:** 1 London’s Air Ambulance, Barts Health NHS Trust, The Royal London Hospital, Whitechapel, London, E1 1FR UK; 2 The Blizzard Institute, Queen Mary University London, Whitechapel, London, UK; 3 Academic Department of Military Emergency Medicine, Royal Centre for Defence Medicine (Research & Clinical Innovation), Birmingham, UK; 4 Department of Emergency Medicine, North Bristol NHS Trust, Westbury On Trym, UK; 5 University Hospitals Bristol & Weston NHS Foundation Trust, Upper Maudlin St , Bristol, UK; 6 NHS Resilience Emergency Capabilities Unit (ECU), Fire Service College, London Rd, Moreton-in-Marsh, UK; 7 London Ambulance Service NHS Trust, London, UK; 8 The Institute of Pre-Hospital Care at London’s Air Ambulance, London, UK; 9 NHS England, London, UK; 10 Department of Trauma and Orthopaedics, Nottingham University Hospitals NHS Trust, Nottingham, UK; 11 University of Plymouth (Faculty of Health) and University Hospitals Plymouth NHS Trust, Plymouth, UK

**Keywords:** Triage, Major incident, Pre-hospital care, Life-saving interventions

## Abstract

**Background:**

Triage at major incidents has traditionally relied on assessing physiology, and in the UK, this role has usually been performed by healthcare responders. However, recent major incidents have shown that this approach, particularly in the initial phases, may be inappropriate due to potential delays in the delivery of key life-saving interventions (LSIs).The Ten Second Triage (TST) tool has been developed to expedite the triage process and the delivery of key LSIs. The aim of this study was to determine the performance characteristics of the TST, including the time to deliver LSIs, in a simulated major incident setting, by both healthcare and non-healthcare responders.

**Methods:**

A prospective observational study was conducted as part of a simulated major incident exercise, incorporating a variety of trauma pathologies. Responders from all three emergency services – Police, Fire, and Ambulance - participated. Eight simulations were conducted, each involving ten responders and a team leader from the respective single service groups. The NHS Major Incident Triage Tool (MITT), the TST alone, and a combination of both were assessed. Quantitative assessments for each simulation included overall triage time, time to delivery of LSI, and accuracy of triage tool categorisation.

**Results:**

Using the TST alone, the mean overall triage time was 377 s (10.2 s per casualty). In comparison, the mean overall triage time of the MITT alone was 1500 s (40.5 s per casualty). However, when the MITT was applied following the TST, it was completed more quickly, with a mean time of 960 s (25.9 s per casualty). When the TST was used, we observed a clinically significant reduction in the time to deliver key LSIs (491 s) compared to the MITT (1377 s). Overall triage accuracy was comparable between TST (80.3%) and MITT (79.3%), and TST performance was similar between healthcare and non-healthcare responders.

**Conclusion:**

This simulation study demonstrated that the TST is a rapid and accurate early scene triage method that enables the timely delivery of key lifesaving interventions. It facilitates the administration of these LSIs faster than existing physiological triage methods. These findings support the NHS England endorsement of the TST.

**Supplementary Information:**

The online version contains supplementary material available at 10.1186/s13049-026-01588-3.

## Background

Triage is a fundamental principle in the effective management of multiple casualty and major incidents. It serves to prioritise casualties based on their clinical acuity and need for treatment, as well as their evacuation needs, thereby initiating and maintaining casualty treatment and ensuring flow to definitive care. However, existing triage tools have demonstrated limited effectiveness in identifying casualties in need of life-saving interventions (LSIs) or in enabling rapid application during the early stages of a multi-casualty or major incident. Traditionally, the triage process has relied on assessing the casualty’s physiological status, such as heart rate and respiratory rate, and is typically performed by trained healthcare responders. Lessons learned from recent major incidents have shown that the use of physiological triage, especially during the initial phases of an incident, may be inappropriate for several reasons, most notably the time required to complete the physiological triage process. It is well documented that if casualties requiring external haemorrhage control and airway management do not receive these critical LSIs within the first few minutes of injury, they are unlikely to survive [1]. When limited responders are faced with significant numbers of casualties, the time required to complete a physiological triage assessment and then deliver key LSIs may be prohibitive, thereby potentially increasing mortality. In the context of a large-scale incident, the use of measured physiological parameters will increase providers’ cognitive burden in an already high-stress situation. Instead, triage tools should be designed to decrease this cognitive load. Previous triage tools have not accounted for the risk of non-compressible haemorrhage from penetrating torso injuries, which has been shown to be the leading cause of preventable death amongst US active shooter victims [2]. An analysis of the patients from the Paris attacks indicated that individuals with central penetrating injuries were typically under-triaged, while those with external compressible injuries were often over-triaged [3].

It has also been observed that challenges exist in measuring physiological parameters early in an incident. This could limit the use of these parameters in adequately validating triage tools intended for early use at the scene. Recent UK inquests into major incidents [4] have highlighted a potential area of concern: existing triage tools often declare casualties who are not breathing as dead, with limited time to assess whether treatment might be feasible, especially when non- healthcare responders arrive first on scene. There are instances where available resources and the mechanism of injury mean that the attempted resuscitation of non-breathing or poorly breathing casualties would be both possible and appropriate [5].

**Fig. 1:**
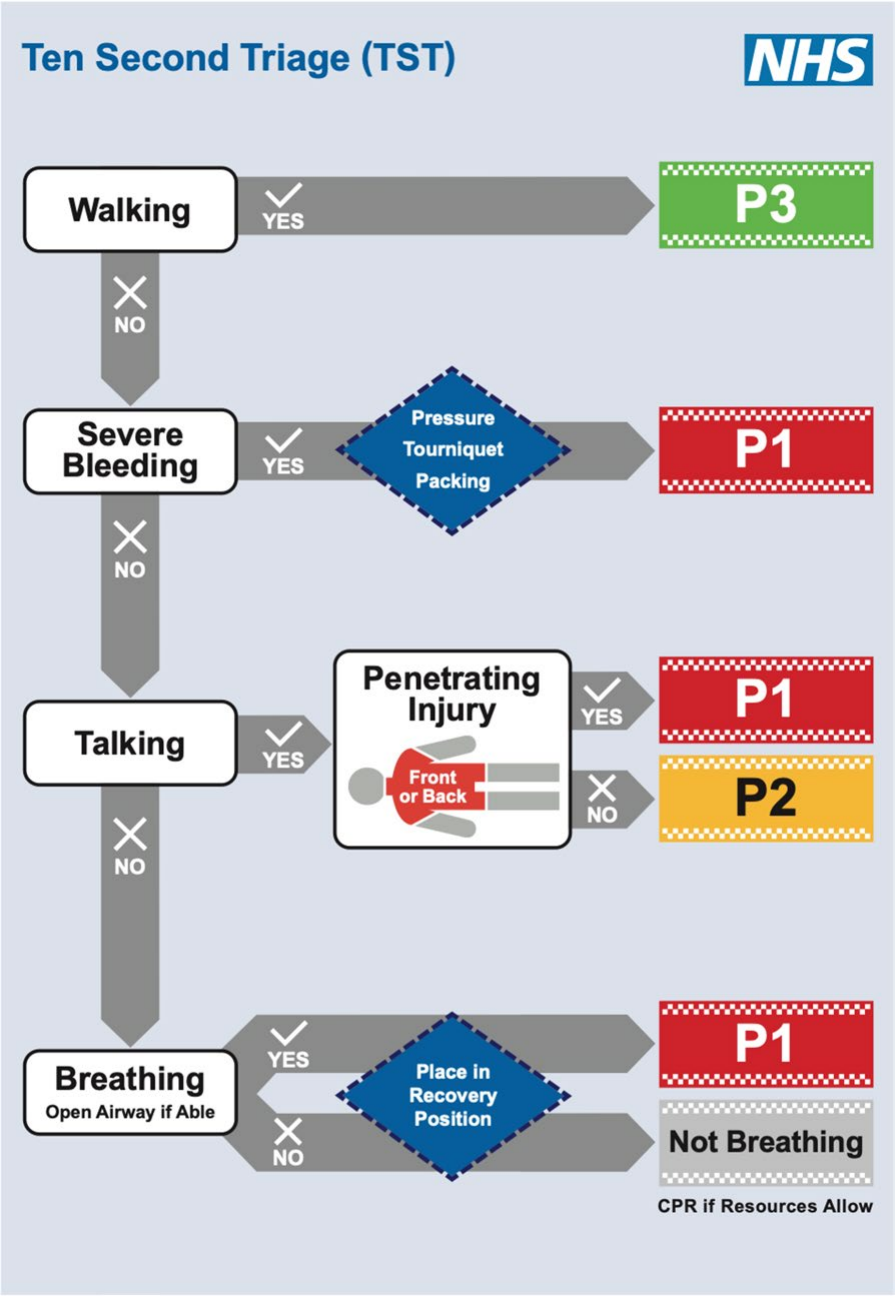
Ten Second Triage Tool flowchart [9]

The Ten Second Triage (TST) tool was developed by an NHS England working group under the direction of the National Strategic Incident Director, with the aim of addressing these issues and implementing solutions. The result was a triage tool that expedited not only the triage process but also the delivery of key LSIs, and could be used by any emergency responder (see Fig. 1). The development of the TST has been described previously [6].

The main aim of this study was to evaluate the performance characteristics of the TST, including the time to delivery of LSIs, in a simulated major incident setting. Secondary aims were to determine its usability among both healthcare and non-healthcare responders, with a mix of participants having varying levels of prior experience.

## Methods

To evaluate the TST, a prospective observational study was conducted at the UK National Ambulance Resilience Unit (NARU) training area.

Participants were drawn from all three emergency services (Police, Fire, and Ambulance) and included both specialist and non-specialist responders. Specialist responders were defined as those equipped with ballistic protection and trained to operate in higher-threat areas. Non-specialist responders did not have ballistic protection or high-threat area training. This is the first time that non-healthcare responders from all emergency services have been trained to triage in the UK.

All participants were volunteers from several regional services across England and did not receive remuneration for their time. Responders represented a range of experience, training, and clinical skills. Participants were assigned to teams of eleven members, including one team leader, within their respective single service groups. Due to the number of Ambulance and Police participants, two groups were formed from each of these services.

To assess TST performance, a simulated major incident was designed, incorporating a range of trauma pathologies including blunt, penetrating, blast, ballistic, and burn injuries. Thirty-three casualties were actors, four of whom were amputees. The remaining four casualties included manikins, with two adult and two paediatric models. Injuries were simulated based on real cases encountered by the authors, (as shown in the table in Additional File 1.) Both live actors and bystanders were briefed on how to act appropriately for each scenario. Each casualty was provided with a laminated injury card (an example is shown in Additional File 2). Exercise faculty members had corresponding injury cards (as shown in Additional File 3). These cards allowed faculty to interject physiological values to candidates using the NHS Major Incident Triage Tool (MITT) [7].

The authors pre-defined the physiological parameters for each casualty’s injury burden, allowing a gold standard triage category to be determined.

Eleven casualties (30.0%) were identified as index cases who required a total of 14 rapid LSIs between them (Table 1). Each casualty had a nearby facilitator acting as a bystander, who was able to observe the timing and efficacy of the LSIs. The LSIs performed for the index casualties included airway opening manoeuvres, tourniquet application, and junctional wound packing. These index casualties were distributed throughout the incident scene, and the positions of all casualties were changed between each run.

**Table 1:** The eleven index casualties required a total of 14 LSIs

Index Number	Injury	Intervention/s required
MDV06	Lower limb amputation	Tourniquet application to leg
MDV09	Head Injury GCS 3 (snoring) & femur fracture	Airway opening manoeuvre
MDV35	Head Injury Impact Brain Apnoea	Airway opening manoeuvre
MBPA14	Stab to groin (femoral artery bleed)	Wound packing/pressure
MBPA 16	Stab to base of neck (jugular vein bleed)	Wound packing/pressure
MCA 22	Paediatric head injury, floppy, apnoeic	Rescue breaths ^1^
MEL 23	Lower leg amputation	Application of Tourniquet to leg
MEL 29	Popliteal and buttock stab wounds	Wound Packing/pressure to buttock Tourniquet application to leg
MEL 31	Brachial artery stab	Tourniquet application to arm
MIED 3	Triple amputation	Tourniquet application × 3 (1 to arm, 2 to legs)
MIED 34	Amputation lower leg	Tourniquet application to leg

Legend: GCS = Glasgow Coma Score; L = Left; R = Right, *MDV* Mechanism Direct Vehicle, *MBPA *Mechanism Bus Penetrating Attack, *MCA *Mechanism Car Attack, *MEL *Mechanism varied External Location, *MIED *Mechanism Improvised Explosive Device

^1^Rescue breaths are taught and prompted only in MITT, not in TST

The time for each run started as all participants entered the simulated scene and began triaging and tagging all casualties, while simultaneously performing any required LSIs. Casualties were tagged using snap wristbands labelled with the TST triage category and colour [8].

A total of eight ‘runs’ of the simulated major incident were conducted.

Run one was performed by Ambulance only, using the MITT as the sole triage tool [7]. This served as the control arm for the study, representing standard ambulance triage practice at a major incident. Runs two to six used only the TST [9] and were conducted by individual emergency service participant groups.

In runs seven and eight, the TST was applied by a larger group of 20 responders from all three emergency services. Run seven was conducted with seven police officers, four firefighters, and nine ambulance responders. Run eight included eight police, four firefighters, and eight ambulance responders. Each of runs seven and eight were followed by the MITT being carried out by the Ambulance Service only (Table 2) to simulate primary triage followed by secondary triage.

**Table 2:** Time to complete scene triage for all 37 casualties

Run No	Participant Group	Triage Tool Used	Time to Complete Primary Triage (secs)	Time to Complete Secondary Triage (secs)	Mean time per casualty (secs)
1	Ambulance (A)	MITT	1500		40.5
2	Ambulance (B)	TST	360		9.7
3	Police (A)	TST	420		11.4
4	Fire	TST	360		9.7
5	Police (B)	TST	300		8.1
6	Ambulance (A)	TST	480		13
7	All Services	TST	300		8.1
	Ambulance (A)	MITT		900	24.3
8	All Services	TST	420		11.4
	Ambulance (B)	MITT		1020	27.6

*Only Run 7 MITT run and Run 8 MITT run had secondary triage applied after the primary triage run, hence these being the only runs with a result in this column*

Regarding the MITT, ambulance responders received a brief 15-min training session delivered by one of the authors prior to run one. All participants received a 30-min training session on the TST, delivered by another author separately prior to run two starting [9].

The quantitative assessments for each run included the overall time to complete the triage of all casualties, time to delivery of LSI, and accuracy of the triage tool categorisation. Additionally, following each run, the participant groups engaged in a qualitative feedback session. The Behavioural Insights Team from the UK Health Security Agency conducted this work, and the output has been described elsewhere [10].

## Results

A total of 55 participants took part (22 from Ambulance, 11 from FRS, and 22 from Police).

A total of 370 casualties were triaged by the TST, the MITT (run one), or a combination of both (runs seven and eight).

### Time to complete scene triage

The overall time to complete the triage process and categorize all casualties ranged from 300 to 1500 s. Using the MITT alone (run one) required the most time at 1500 s, a mean time of 40.5 s per casualty. In contrast, when using the TST alone (runs two to six), the range was 300 to 480 s, corresponding to 8–13 s per casualty. When all runs utilizing the MITT (runs one, seven, and eight) are considered, the mean time to complete the triage process was 1140 s, equating to 31 s per casualty. In contrast, for the TST (runs two to eight), the mean time to complete the triage process was 377 s, equating to 10 s per casualty.

When all participants were considered, both Fire and Police completed the triage process more quickly (mean time 360 s, or 10 s per casualty), compared to Ambulance personnel (mean time 420 s, or 11 s per casualty). A detailed breakdown of the results is presented in Table 2.

In runs seven and eight, there was a mean reduction of 540 s to complete the MITT compared to run one. This corresponds to a mean reduction of 15 s per casualty.

### Time to delivery of LSI

For each run, the time required to perform the necessary LSIs for the index casualties was measured.

A clinically significant reduction in time to LSI was observed when the TST was used, as compared to the MITT (see Table 3). (mean 491 s vs 1377 s). This is the length of time it took for the last index casualty to receive the required LSIs.

**Table 3:** Longest times taken to perform the required LSIs for the index casualties

Run No	Participant Group	Triage Tool Used	Longest time to delivery of LSI (secs)
1	Ambulance (A)	MITT	1377
2	Ambulance (B)	TST	340
3	Police (A)	TST	320
4	Fire	TST	416
5	Police (B)	TST	185
6	Ambulance (A)	TST	409
7	All Services	TST	233
8	All Services	TST	158

LSIs were administered to all index casualties in the shortest period by police (253 secs), followed by Ambulance personnel (374 secs) and Fire personnel (416 secs).

Timings for LSIs have not been provided for the use of the MITT in runs 7 and 8 because they were performed during the TST for these runs.

It should also be noted that runs seven and eight had more responders (20 compared to 10 for all other runs) for TST, resulting in a more rapid completion time for all LSIs.

### Accuracy of triage tool categorization

A comparable performance was observed in overall triage accuracy for both TST and MITT (80.3% and 79.3%, respectively), with similar rates of under-triage and over-triage.

Full data on the accuracy of the triage tools is provided in Table 4.

**Table 4:** Accuracy of triage tool categorisation for each round

				**Correct Triage**		**Over-triage**		**Under-triage**		**Missed casualties**	
**Run No**	**Participant Group**	**Triage Tool Used**	**Casualties**	**N**	**%**	**N**	**%**	**N**	**%**	**N**	**%**
1	Ambulance (A)	MITT	37	27	73.0%	4	10.8%	3	8.1%	3	8.1%
2	Ambulance (B)	TST	37	33	89.2%	4	10.8%	0			
3	Police (A)	TST	37	29	78.4%	5	13.5%	3	8.1%		
4	Fire	TST	37	26	70.3%	7	18.9%	4	10.8%		
5	Police (B)	TST	37	27	73.0%	5	13.5%	4	10.8%	1	2.7%
6	Ambulance (A)	TST	37	32	86.5%	5	13.5%	0			
7	All Services	TST	37	33	89.2%	3	8.1%	1	2.7%		
	Ambulance (A)	MITT	37	30	81.1%	6	16.2%	1	2.7%		
8	All Services	TST	37	28	75.7%	5	13.5%	4	10.8%		
	Ambulance (B)	MITT	37	31	83.8%	4	10.8%	2	5.4%		
All runs TST (2–8)			259	208	80.3%	34	13.1%	16	6.2%	1	0.4%
All runs MITT (1,7,8)			111	88	79.3%	14	12.6%	6	5.4%	3	2.7%

*“Missed casualties” are defined as those who were neither tagged nor counted, and who were not included in the final numbers reported to the ambulance team leader at the end of each run. NB: Participants were unaware of the total number of casualties present*

## Discussion

In this study, we utilised a simulated major incident to successfully demonstrate the practical utility of the TST in the early phases of a major incident. We have demonstrated its ability to be used by all emergency services personnel, not just healthcare responders and shown that its use reduces the time to delivery of key LSIs whilst maintaining sufficient overall accuracy.

The period following the incident occurring and prior to the provision of any on scene care has previously been referred to as the ‘therapeutic vacuum’ [11] and has more recently been termed the ‘Care Gap’ by Sir John Saunders, chairman of the Manchester Arena Inquiry [4].

This period requires both the rapid implementation of LSIs and then rapid movement of casualties to facilitate delivery of definitive care. These actions are essential in order to fulfil the Joint Emergency Service Interoperability Principles (JESIP) [12] of “saving life and reducing harm.”

### Delivery of LSIs

The TST flowchart prompts the control of external catastrophic haemorrhage and maintenance of airway patency, corresponding to the care provided during buddy-buddy aid on the battlefield, which should be administered by any first responder who is capable. By enabling the provision of these early LSIs, we aim to reduce mortality and morbidity from potentially salvageable injuries and help close the care gap.

### Use by all responders

The ability of TST to be used by all responders is particularly important in the early stages of an incident when the responder-to-casualty ratio is low, as was summarized by Sir John Saunders, who noted, “*if all those present in the city room on the night of the attack had been trained in Ten Second Triage, triage would have been much more efficient”* [4].

During the rapid, multi-agency response to many major incidents, particularly those influenced by terrorism or with public disorder, where there is a high likelihood of increased threat within the initial scene, conventional healthcare responders may be unable to access the site promptly, In these circumstances, initial triage would likely be performed by non-healthcare responders [13, 14].

Our findings demonstrate that TST can be delivered effectively by both healthcare and non-healthcare responders, and that by using the TST, they are able to deliver key LSIs more effectively and efficiently than when the MITT is used. These findings are consistent with non-healthcare responders performing triage as reported in the literature [15, 16].

We have also seen that using a unified tool facilitates effective multi-agency work, as it provides a common language and improves situational understanding. This enhanced efficacy is echoed in previously published personal reflections from participants [10].

### Time to triage delivery

In this study, we observed that for all responders, the mean time required to use the TST was significantly shorter (10 s per casualty) compared to the mean time for all MITT runs combined (31 s per casualty). Unexpectedly, we also found that the time taken to use the MITT decreased when it was preceded by the TST (1500 s vs 960 s).

While many studies report triage tool performance in terms of accuracy, few provide information on the time taken to complete the process. Those that do report timing show a wide range; the fastest is the SMART triage method (29 s per casualty), while the slowest is the Sacco Triage Method (71 s per casualty) [17, 18].

The TST outperforms both methods in speed while maintaining a comparably high accuracy for triage categorisation (80.3%) to that of the MITT (79.3%).

We observed in this study that healthcare personnel took approximately 2 s longer to perform the TST than non-healthcare professionals. This may be related to healthcare personnel being accustomed to performing physiological triage, which takes longer, and being taught to do so in pairs. They also may have a tendancy to use clinical acumen as well as having more intervention options available, and so take longer to focus on the key questions in TST, perform the LSIs and move onto the next casualty. On an individual casualty basis, this time difference is unlikely to be clinically significant. However, when large volumes of casualties are encountered, it may then become significant. Longer triage times resulted in increased delays in delivering key LSIs to index casualties. In large incidents with higher numbers of casualties requiring LSIs, this may well impact critical mortality rates (i.e., deaths among potentially salvageable casualties).

### Limitations

While our use of a simulated major incident setting allows for the assessment of the TST and MITT’s performance in more ways than a trauma registry analysis (e.g. speed of LSI delivery, time to complete triage, and usability), we acknowledge several limitations.

Firstly, each triage run was conducted with eleven participants: ten performing triage and one team leader. All entered the major incident scene at the same time to conduct triage and deliver LSIs simultaneously. The nature of emergency services response to an incident makes this scenario unlikely to be truly representative of a real-life event. In reality, a more staggered or at least clustered approach to the arrival of resources is more likely.

Secondly, regarding triage categorisation, these were determined a priori for the MITT. based on what the authors believed the casualties’ level of physiological derangement would be as matched with their respective injury burdens. We acknowledge that these categorisations, while determined through experience and derived from past cases, may not be entirely representative of all casualties.

The TST triage categories were based on the injury and the actual clinical presentation. The casualty scenarios presented to participants included descriptions such as walking, talking breathing or not breathing. This was carried out according to the directions given by the authors to the casualty actors. We therefore acknowledge that the limitations of live actors meant the “not breathing” casualties required facilitator input, to provide the “not breathing” finding when airway opening and breathing assessments were performed by responders on actors who were designated as “unresponsive”.

Thirdly, we acknowledge that by standardising the casualties within each run, participants who performed repeat runs (e.g., Ambulance and Police A and B groups) may have become familiar with the incident scene, which may have led to an improvement in triage tool performance, as measured by time to LSI delivery and time to triage all casualties. To address this, casualties did not remain static and were moved around the incident scene between runs. Additionally, it was unlikely that the same responder would end up in the same section of the incident during each run in which they participated.

Lastly, we acknowledge that other triage tools (e.g., RAMP and Careflight), designed for rapid use at incidents, have been described elsewhere [19, 20]. The aim of this study was not to conduct a comparative analysis to determine the optimal triage tool. It was to evaluate specifically the performance of the TST, a novel triage tool that does not rely on any physiological measurements, including even minimal assessments such as palpation of the radial pulse, in comparison to the MITT, and its potential for use by both healthcare and non-healthcare responders. Therefore, no other triage tools were considered in the study design.

## Conclusion

A key tenet of major incident triage is that it must be rapid, reliable, and reproducible.

This holds true irrespective of the provider using the system. Furthermore, to reduce both morbidity and mortality, casualties requiring LSIs must receive them in a timely fashion.

In this simulated observational study, we have successfully demonstrated that the TST not only meets the necessary requirements of a triage tool, but also allows for the delivery of LSIs in a more expedient manner than existing methods of physiological triage.

Key to this is its ability to be used effectively by non-healthcare responders, thereby maximizing the number of responders able to deliver essential LSIs at the scene of a major incident, and help to reduce preventable deaths. These findings support NHS England’s endorsement of the TST for use by all emergency services, as well as the associated endorsement by the National Fire Chiefs’ Council and the National Police Chiefs’ Council.

## Supplementary Information

Additional file 1. Simulated injury patterns for all casualties.

Additional file 2. Example of a casualty laminated card.

Additional file 3. Example of an instructor laminated card.

## Data Availability

All relevant data is summarised either within the tables in the manuscript itself or in the Additional Files provided. The original datasets used and/or analysed during the current study are available from the corresponding author on reasonable request.

## Abbreviations

LSIs: Life-Saving Interventions
TST: Ten-Second Triage
MITT: Major Incident Triage Tool
NHS: National Health Service
NHSE: National Health Service England
NARU: National Ambulance Resilience Unit
GCS: Glasgow Coma Scale
RAMP: Rapid Assessment and Medical Prioritisation
