# Supplementary material for: The Ten Second Triage Tool – a multi-disciplinary simulation-based field test to determine its speed, accuracy and practical on scene application
Source: Scand J Trauma Resusc Emerg Med. 2026 Mar 23;34:82. doi: 10.1186/s13049-026-01588-3 (PMC13130538; doi:10.1186/s13049-026-01588-3)
Supplement: Supplementary file 1 — Additional file 1. Simulated injury patterns for all casualties. [file 13049_2026_1588_MOESM1_ESM.docx]

**Additional File 1: Simulated injury patterns for all casualties**

**TST Final Field Test Casualties Summary Table**

Scenario: Deliberate vehicle attack, bus occupants subject to Marauding Terrorist Attack with bladed weapons, car subject to petrol bomb attack and victims of Person Borne Improvised Explosive Device (PBIED) in enclosed space.

- Casualties are grouped into
  - MDV: Pedestrians from Deliberate Vehicle Attack, Blunt trauma
  - MBPA: Passengers on bus, penetrating attack
  - MCA: Car occupants, blunt trauma and burns
  - MEL: Assorted others in proximity to above, can be moved to vary scenario
  - MIED: Number of patients from PBIED in enclosed space
- Casualties requiring a life saving intervention (LSI) are flagged as an “index casualty’ with the required intervention noted in the second column.
- The table is a brief overview of the casualty details and key presentation points.

| **Number** | **Index pt and intervention** | **Type** | **Injuries** |
| --- | --- | --- | --- |
| **MDV-01** | n/a | Live Patient | Obvious closed ankle & wrist #  Well |
| **MDV-02** | n/a | Live Patient | Bilateral open fractures to feet  Well |
| **MDV-03** | n/a | Mannequin  (Baby in Pram) | Baby  Peri-arrest.  Bleeding from mouth and nose  Bradycardic & low respirations for age. |
| **MDV-04** | n/a | Live Patient  (Mother of above) | Open L femur fracture  Closed R femur fracture  Pain, pale, otherwise well |
| **MDV-05** | n/a | Live Patient | Left chest reduced air entry  Pelvic fracture (splayed legs)  No radial, just carotid |
| **MDV-06** | **Index <C>**  CAT to R leg | Live Patient | Mangled Right lower leg ongoing bleeding  Left lower leg fracture  Pale sweaty agitated |
| **MDV-07** | n/a | Live Patient | Open book pelvic fracture  Muttering re pain in groin and back (not talking normally)  No radial, carotid only, sweaty and pale  Fast shallow breathing  Bleeding PU / PR |
| **MDV-08** | (Open airway but still not breathing ) | Live Patient | Elderly male. Hit by van  Multiple open fractures  Skull fracture  Blood in airway  Not breathing |
| **MDV-09** | **Index A**  jaw thrust and recovery position | Live Patient | Head injury and femurs  R thigh fracture (deformed)  Unresponsive, snoring  Bleeding nose, ears |
| **MDV-27** | N/a | Child Mannequin | Polytrauma  Large head wound. Blood mouth, nose, ears  Bruising chest and abdomen  Deformed right thigh and splayed legs  Not breathing |
| **MDV-28** | n/a | Live Patient | Awake and screaming  Closed R Tib/Fib &  Open L Ankle |
| **MDV35** | **Index A**  Jawthrust and recovery position | Live patient | **Head injury -**  Ped vs vehicle. Impact Brain Apneoa  Bruising forehead, occipital haematoma  Unresponsive  Obstructed airway, no resp effort  If jaw thrust / npas rapidly then starts breathing … if >8 mins in then needs some ventilation then will start breathing  if >12 mins dead |
| **MBPA-10** | n/a | Live Patient | Slash wounds to face,  & R hand - fingers partially amputated  ‘Oozing’ |
| **MBPA-11** | n/a | Live Patient | Penetrating wound with pneumothorax R chest  Evolving haemothorax and pneumothorax  R ACF slash wound ‘oozing’  Rapid RR with increased work of breathing (not shallow) |
| **MBPA-12** | n/a | Live Patient | Multiple slash wounds to arms and face  oozing no <C>  Sobbing, a bit pale. Well but wont walk |
| **MBPA-13** | n/a | Live Patient | Stab wound to abdomen with evisceration of small bowel, bradycardic  Vagal not bleeding |
| **MBPA-14** | **Index <C>**  Groin Packing | Live Patient | Groin wound with femoral artery bleed  Profunda femoris bleeding  Too high for tourniquet  <C>  No radial, pale  Rapid shallow breathing (air hunger) |
| **MBPA-15** | n/a | Live Patient | Stab wounds to both legs, no <C>  Slash wounds back of both upper legs  Cant walk because injury at back of legs painful but looks and is well |
| **MBPA-16** | **Index <C>**  Neck packing and pressure | Live Patient | Slash wound to neck with <C>  Pale, not saying much, rapid shallow breathing |
| **MBPA 17** | n/a | Live Patient | Stab R Groin, ACF, Axilla, Neck  No more <C>  Pale, unresponsive  Not breathing |
| **MBPA-18** | n/a | Live Patient or Mannequin | Slash wound to neck,  Partial decapitation  Unresponsive. Not breathing |
| **MBPA-19** | ***Needs minor haem control but not Index*** | Live Patient | Slash wound to neck with ooze - not cat haem |
| **MBPA 36** | n/a | Live patient | **Stab R lower thoracic area**  Bleeding hemiazygous vein.  Awake. pale, sweating – initially pulses present weakly, staring and not talking normally  Shocked not tachycardiac  Then no distal pulses, low bp becomes unrecordable. Complaining of thirst progressing to altered sensorium.  Non compressible haem – needs identification as P1 and extrication rapidly |
| **MCA-20** | n/a | Live Patient or SimMan | Driver, >80% BSA burns: face arms & legs ,  ‘Moaning words’ not talking normally  Airway noisy, stridor, breathing rapid |
| **MCA-21** | n/a | Live | Passenger of car, burns to hands  ‘slightly rapid breathing ’  Coughing and dyspnoiec |
| **MCA-22** | **Index – A**  and **rescue breaths** | Mannequin | Paediatric head injury female toddler  Abrasion forehead  Moaning, floppy  Apnoieic with occluded airway  Rapid breathing after rescue breaths |
| **MEL-23** | **Index <C>**  CAT R leg | Amputee | Amputated R lower leg bleeding+++  and open fracture. |
| **MEL-24** | n/a | Mannequin | GSW chest and head  Multiple  Unresponsive, not breathing |
| **MEL-25** | n/a | Live | Heavily pregnant female, stab wound to lower abdomen (RIF) & pain  Syncopal if standing  Wont leave child  Uterine injury |
| **MEL-26** | n/a | Mannequin (Child) | Pre-school child, quiet,  covered in blood spatter.  Moaning, won’t walk  No injuries |
| **MEL-29** | **Index <C>**  CAT L leg & buttock packing and pressure | Live patient | Stab L buttock  L popliteal fossa  Pale – agitated  No radial |
| **MEL 30** | n/a | Live Patient | Slash face – talking, walking with assistance  Blood constantly dripping from face into mouth |
| **MEL 31** | **Index <C>**  CAT arm | Live Patient | Slash ACF – brachial artery injury.  Severe bleeding  V pale, rousable to pain  Rapid resp rate |
| **MIED 32** | **Index <C>**  <CAT> x 3 | Triple amp live pt | Muttering – words not quite sensible  Bleeding from three limbs |
| **MIED 33** | n/a | Live Patient | Bilateral Closed femur fractures,  Blast injury, multiple shrapnel to torso.  Talking with difficulty (not normally) |
| **MIED 34** | **Index <C>**  CAT R leg | Live Patient | Traumatic amputation R lower leg  Pale, sweaty  Quiet but in pain++ and distressed |
| **MIED 37** | n/a | Mannequin / sand dummy | **Protagonist**  Dismembered protagonist  Extensive facial destruction  Bilat leg amputation: high L, mid R.  Male v pale, unresponsive  Not breathing |
