# Supplementary material for: The Ten Second Triage Tool – a multi-disciplinary simulation-based field test to determine its speed, accuracy and practical on scene application
Source: Scand J Trauma Resusc Emerg Med. 2026 Mar 23;34:82. doi: 10.1186/s13049-026-01588-3 (PMC13130538; doi:10.1186/s13049-026-01588-3)
Supplement: Supplementary file 2 — Additional file 2. Example of a casualty laminated card. [file 13049_2026_1588_MOESM2_ESM.docx]

**MBPA-16**


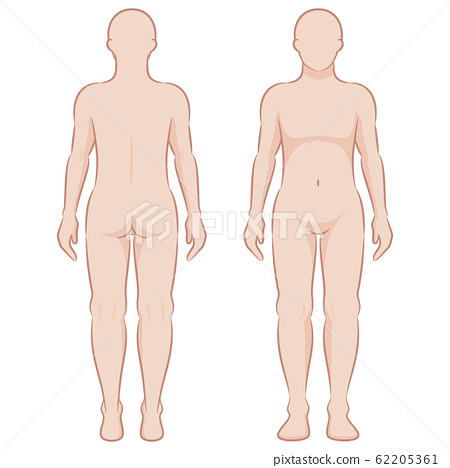


**What you see and hear:**

- **<C> neck wound**
- **Male, very pale**
- **Quiet not talking normally**
- **Rapid & shallow**
- **Slash wound to neck bleeding profusely**
