# Supplementary material for: The Ten Second Triage Tool – a multi-disciplinary simulation-based field test to determine its speed, accuracy and practical on scene application
Source: Scand J Trauma Resusc Emerg Med. 2026 Mar 23;34:82. doi: 10.1186/s13049-026-01588-3 (PMC13130538; doi:10.1186/s13049-026-01588-3)
Supplement: Supplementary file 3 — Additional file 3. Example of an instructor laminated card. [file 13049_2026_1588_MOESM3_ESM.docx]

**Casualty Number: MBPA-16**

**Faculty Sheet**


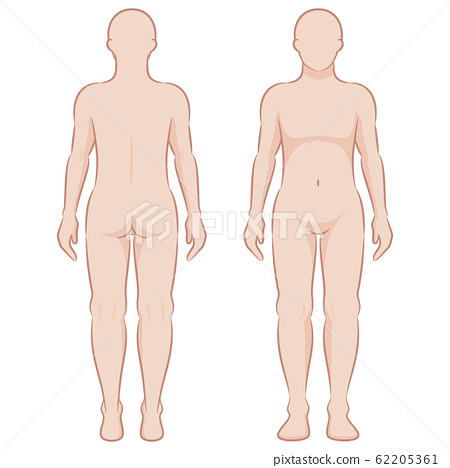


| **Counted in:** | | | | | |
| --- | --- | --- | --- | --- | --- |
|  | **10 seconds** | **15 seconds** | **20 seconds** | **30 seconds** | **60 seconds** |
| **Resp Rate** | **2** | **3** | **4** | **6** | **12** |
| **Pulse Rate** | **10** | **15** | **20** | **30** | **60** |

**<C> [C] neck**

**Appearance Male. very pale**

**Level of response Responsive to Pain**

**Breathing quality Slow & shallow**

**Any other visual cues Slash wound to neck, with profuse bleeding**

**Interventions: Tourniquet / Haemostatic/ Airway adjunct / Recovery Position / Declared Dead**

|  | **Run 1** | **Run 2** | **Run 3** | **Run 4** | **Run 5** |
| --- | --- | --- | --- | --- | --- |
| **Time to Tag from arrival** |  |  |  |  |  |
| **Team (A or B)** |  |  |  |  |  |
| **Interventions** |  |  |  |  |  |
| **Triage Category** |  |  |  |  |  |
